# Supplementary material for: Host CDK-1 and formin mediate microvillar effacement induced by enterohemorrhagic Escherichia coli
Source: Nat Commun. 2021 Jan 4;12:90. doi: 10.1038/s41467-020-20355-1 (PMC7782584; doi:10.1038/s41467-020-20355-1)
Supplement: Supplementary file 1 — Supplementary Information [file 41467_2020_20355_MOESM1_ESM.pdf]

Supplemental Information

Supplementary Tables

Supplementary Table 1. *C. elegans* Strains.

DESCRIPTION	SOURCE	IDENTIFIER
C. elegans: wild type	Caenorhabditis Genetics Center (CGC)[3]	Strain: N2
C. elegans: unc-119(ed3);dkIs247[act-5p::mCherry::HA::act-5;Cb.unc-119(+)]	[4]	Strain: GK454
C. elegans: unc-119(ed4);upsIs3[cyk-1p::cyk-1::gfp;unc-119(+)]	[5]	Strain: DWP13
C. elegans: unc-119(ed3);dotSi100[T06E6.2/cyb-3;unc-119(+)]	[6]	Strain: JNC100
C. elegans: unc-119(ed3);dkI5166[opt-2p::GFP::pgp-1;unc-119(+)]	[7]	Strain: GK288
C. elegans: kcIs6[IFB-2::CFP];caIs[ges-1::YFP::ACT-5]	[8]	Strain: ERT38
Ex[gfp::act-5, pRF4]	Junho Lee (Seoul National University, Korea)[9]	Strain: GFP::ACT-5
C. elegans: cdk-1(ne2257)	[10]	Strain: WM99
C. elegans: unc-119(ed3);wfIs52[app-1p::mCherry:histone H2B::unc-54]	[11]	Strain: YQ203
C. elegans: unc-119(ed3);dotSi100[T06E6.2;unc-119(+)];dkIs247[act-5p:: mCherry::HA::act-5;Cb.unc-119(+)]	this study	Strain: YQ387
C. elegans: unc-119(ed3);wfEx338[app-1p::LifeAct::GFP::unc-54;unc-119(+)];dkIs247[act-5p::mCherry::HA::act-5;Cb.unc-119(+)]	this study	Strain: YQ388
C. elegans: pfn-1(ok808); unc-119(ed3);dkIs247[act-5p::mCherry::HA::act-5;Cb.unc-119(+)]	this study	Strain: YQ389
C. elegans: unc-119(ed4);upsIs3[cyk-1p::cyk-1::gfp;unc-119(+)];dkIs247[act-5p::mCherry::HA::act-5;Cb.unc-119(+)]	this study	Strain: YQ420
C. elegans: cdk-1(ne2257);unc-119(ed3);dkIs247[act-5p::mCherry::HA::act-5;Cb.unc-119(+)]	this study	Strain: YQ422
C. elegans: unc-119(ed3);dkIs247[act-5p:: mCherry::HA::act-5;Cb.unc-119(+);wfEx388[app-1p::cyk-1::GFP::unc-54]	this study	Strain: YQ486
C. elegans: unc-119(ed3);dkIs247[act-5p:: mCherry::HA::act-5;Cb.unc-119(+);wfEx392[app-1p::cyk-1^{T611A}::GFP::unc-54]	this study	Strain: YQ487

C. elegans: unc-119(ed3);dkIs247[act-5p::mCherry::HA::act-5;Cb.unc-119(+)];wfEx391[app-1p::cyk-1^{T1232A}::GFP::unc-54]	this study	Strain: YQ488
C. elegans: unc-119(ed3);dkIs247[act-5p::mCherry::HA::act-5;Cb.unc-119(+)];wfEx390[app-1p::cyk-1^{T1273A}::GFP::unc-54]	this study	Strain: YQ489
C. elegans: unc-119(ed3);dkIs247[act-5p::mCherry::HA::act-5;Cb.unc-119(+)];wfEx396[app-1p::cyk-1^{T1273E}::GFP::unc-54]	this study	Strain: YQ490
C. elegans: unc-119(ed4);upsIs3[cyk-1p::cyk-1::gfp;unc-119(+)];dkIs247[act-5p::mCherry::HA::act-5;Cb.unc-119(+)];wfEx404[app-1p::cdk-1::bfp::unc-54]	this study	Strain: YQ494
C. elegans: unc-119(ed3);dkIs247[act-5p::mCherry::HA::act-5;Cb.unc-119(+);wfEx388[app-1p::cyk-1::GFP::unc-54]	this study	Strain: YQ525
C. elegans: unc-119(ed3);dkIs247[act-5p::mCherry::HA::act-5;Cb.unc-119(+)];wfEx390[app-1p::cyk-1^{T1273A}::GFP::unc-54]	this study	Strain: YQ526
C. elegans: unc-119(ed3);dkIs247[act-5p::mCherry::HA::act-5;Cb.unc-119(+)];wfEx396[app-1p::cyk-1^{T1273E}::GFP::unc-54]	this study	Strain: YQ527

Supplementary Table 2. Bacterial Strains.

DESCRIPTION	SOURCE	IDENTIFIER
E. coli. Uracil auxotrophy, standard *C. elegans* laboratory food source.	Caenorhabditis Genetics Center (CGC)[3]	Strain: OP50
E. coli O157:H7 isolated from raw hamburger meat implicated in hemorrhagic colitis outbreak.	Bioresource Collection and Research Center (BCRC)[12]	Strain: EDL933
E. coli O157:H7 isolated from human stool	Bioresource Collection and Research Center (BCRC)[13]	Strain: HER1266
E. coli O157:H7 isolated from human faeces; does not produce either Shiga-like toxin 1 or 2 and does not process the genes for these toxins	Bioresource Collection and Research Center (BCRC)[14]	Strain: ATCC43888
E. coli HT115 with L4440, an empty vector, used as negative control of RNAi	Caenorhabditis Genetics Center (CGC)[15]	Strain: HT115
E. coli EDL933:*Δler*, EDL933 isogenic mutant with *ler* deletion	this study	Strain: YQ53
E. coli EDL933:*ΔespA*, EDL933 isogenic mutant with *espA* deletion	this study	Strain: YQ495
E. coli EDL933:*ΔespB*, EDL933 isogenic mutant with *espB* deletion	this study	Strain: YQ50
E. coli EDL933:*ΔespD*, EDL933 isogenic mutant with *espD* deletion	this study	Strain: YQ51
E. coli EDL933:*ΔescN*, EDL933 isogenic mutant with *escN* deletion	this study	Strain: YQ57
E. coli EDL933:*Δeae*, EDL933 isogenic mutant with *eae* deletion	this study	Strain: YQ62
E. coli EDL933:*ΔespF*, EDL933 isogenic mutant with *espF* deletion	this study	Strain: YQ496
E. coli EDL933:*ΔespFu*, EDL933 isogenic mutant with *espFu* deletion	this study	Strain: YQ497
E. coli EDL933:*ΔespG*, EDL933 isogenic mutant with *espG* deletion	this study	Strain: YQ498
E. coli EDL933:*ΔespH*, EDL933 isogenic mutant with *espH* deletion	this study	Strain: YQ499
E. coli EDL933:*ΔespM*, EDL933 isogenic mutant with *espM* deletion	this study	Strain: YQ500
E. coli EDL933:*Δmap*, EDL933 isogenic mutant with *map* deletion	this study	Strain: YQ501
E. coli EDL933:*Δtir*, EDL933 isogenic mutant with *tir* deletion	this study	Strain: YQ52
E. coli EDL933:*ΔhlyA*, EDL933 isogenic mutant with *hlyA* deletion	this study	Strain: YQ56

E. coli EDL933:*ΔhlyE*, EDL933 isogenic mutant with *hlyE* deletion	this study	Strain: YQ113
E. coli EDL933:*Δstx1*, EDL933 isogenic mutant with *stx1*deletion	[1]	Strain: YQ58
E. coli EDL933:*Δstx2*, EDL933 isogenic mutant with *stx2* deletion	[1]	Strain: YQ59
E. coli EDL933:*ΔrfaD*, EDL933 isogenic mutant with *rfaD* deletion	[16]	Strain: YQ217
E. coli OP50-GFP, *E. coli* OP50 transformed with pFPV25.1; Am^r	[1]	Strain: YQ368
E. coli EDL933-GFP, EDL933 transformed with pFPV25.1; Am^r	[1]	Strain: YQ369

Supplementary Table 3. Plasmids.

DESCRIPTION	SOURCE	IDENTIFIER
Plasmid: an empty vector was used as negative control of RNAi	Caenorhabditis Genetics Center (CGC)[15]	L4440
Plasmid: *cyk-1p::cyk-1::GFP*	[5]	pRS315
Plasmid: *app-1p::LifeAct::GFP::unc-54*	this study	pWF338
Plasmid: *app-1p::cyk-1*^{T1273A}*::GFP::unc-54*	this study	pWF390
Plasmid: *app-1p::cyk-1*^{T1223A}*::GFP::unc-54*	this study	pWF391
Plasmid: *app-1p::cyk-1*^{T611A}*::GFP::unc-54*	this study	pWF392
Plasmid: *app-1p::cyk-1*^{T1273E}*::GFP::unc-54*	this study	pWF396
Plasmid: *app-1p::cdk-1::bfp::unc-54*	this study	pWF404
Plasmid: shCCNB3	this study	pWF424

Supplementary Table 4. Primers.

DESCRIPTION	SEQUENCES	SEQUENCES
cyb-3 mRNA	F:CTCGTGCTATCCTCATTGACTG	R:CATGCAAGCTTCTGAATGGTG
cyk-1::gfp mRNA	F:TCCAGTTACGAATATCAATGG	R:CAACAAGAATTGGGACAACT
mCherry::act-5 mRNA	F:CAACGTCAACATCAAGTTGGA	R:GCGGCGATTTCTTCTTCCAT
gfp::act-5 mRNA	F:GCCCTTTCGAAAGATCCCAA	R:GCGGCGATTTCTTCTTCCAT
yfp::act-5 mRNA	F:ACTACCTGAGCTACCAGTCC	R:GCGGCGATTTCTTCTTCCAT
cyd-1 mRNA	F:TCCAGTTGGAAGTGAACCTTCT	R:GGTTCCGTCTTCTCAGCATC
cdk-4 mRNA	F:GGAAAAGACTATGCACTCAAGCA	R:ATGAGCTTTTCGTGCCAGAT
cye-1 mRNA	F:ACATGACGAACTGCGTGAAC	R:TGCAGCTGGACCTTCCTTAG
cdk-2 mRNA	F:GGTGCTCTCTCTCATCGCTAC	R:GGTGGCGTCCTGTTCTGC
cya-2 mRNA	F:CGATGAGGATCGCAGTAAAA	R:CGATGAAGTCGAACTTTCCAA
gpd-3 mRNA	F:GACTATGTCGTTGAGTCCACC	R:ATGATCGTACTTCTCGTGGTT G
CCNB3 mRNA	F:CATCATGACCCATCTGAAAAGAC GG	R:AGTGAGATCTTCAAAAGCTG ATCTCTT
DIAPH1 mRNA	F:TCTGCCCAGACCAAGACTTC	R:TTTTTCTTTTGCACAGATTT CTTTT
DIAPH2 mRNA	F:AAGTTTGTGGAAAAGATGACCAG	R:TCATAGAGCTTCATCATGTT GTTGT
DIAPH3 mRNA	F:TGATATTGGAAGTAGATGAAACAC G	R:CAGGAAGATGCTTTATTAAG TTCTG A
GAPDH mRNA	F:ATCATCAGCAATGCCTCC	R:GGATGATGTTCTGGAGAG

Supplemental Figures

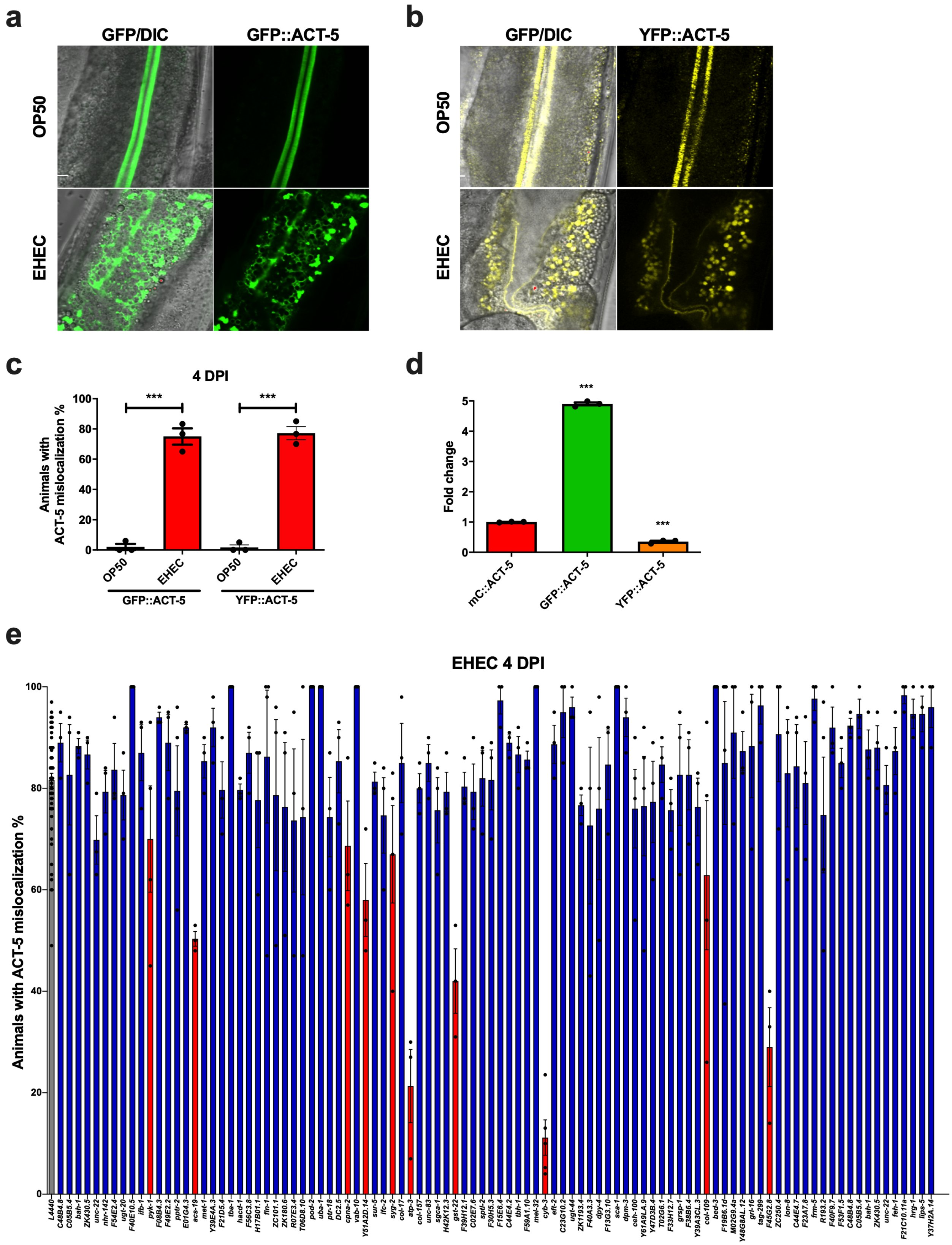

Fig. S1. EHEC induced mislocalization of ACT-5s, and the RNAi screening results. (a) Confocal images of the GFP::ACT-5 animals fed with OP50 or EHEC for 4 days are shown. GFP/DIC are the merged images of the GFP images overlaid with the differential interference contrast (DIC) images. The scale bars represent 5 μm. **(b)** Confocal images of the YFP::ACT-5 animals fed with OP50 or EHEC for 4 days are shown. YFP/DIC are the merged images of the YFP images overlaid with the DIC images. The scale bars represent 5 μm. **(c)** The quantitative results for the ectopic localization of fluorescent proteins in the GFP::ACT-5 worms (in S1a; n=106, N=3, P=0.0002 compared to the OP50 control group by unpaired *t*-test (two-tailed)) and YFP::ACT-5 worms (in S1b; n=100, N=3, P<0.0001 compared to the OP50 control group by unpaired *t*-test (two-tailed)) fed with OP50 or EHEC. **(d)** qRT-PCR analysis of mRNA levels of the three *act-5* transgenes. ***, P<0.001 compared to the mC::ACT-5 group by unpaired *t*-test (two-tailed) **(e)** The results of the RNAi screen for the host factors involved in EHEC-induced ACT-5 milocalization phenotype. All representative confocal images were performed independently for three times. All quantitative data are presented as mean±SEM, and each dot represented an independent result in the bar chart. All data statistics based on: * P<0.05, ** P<0.01, and *** P<0.001 by unpaired *t*-test (two-tailed).

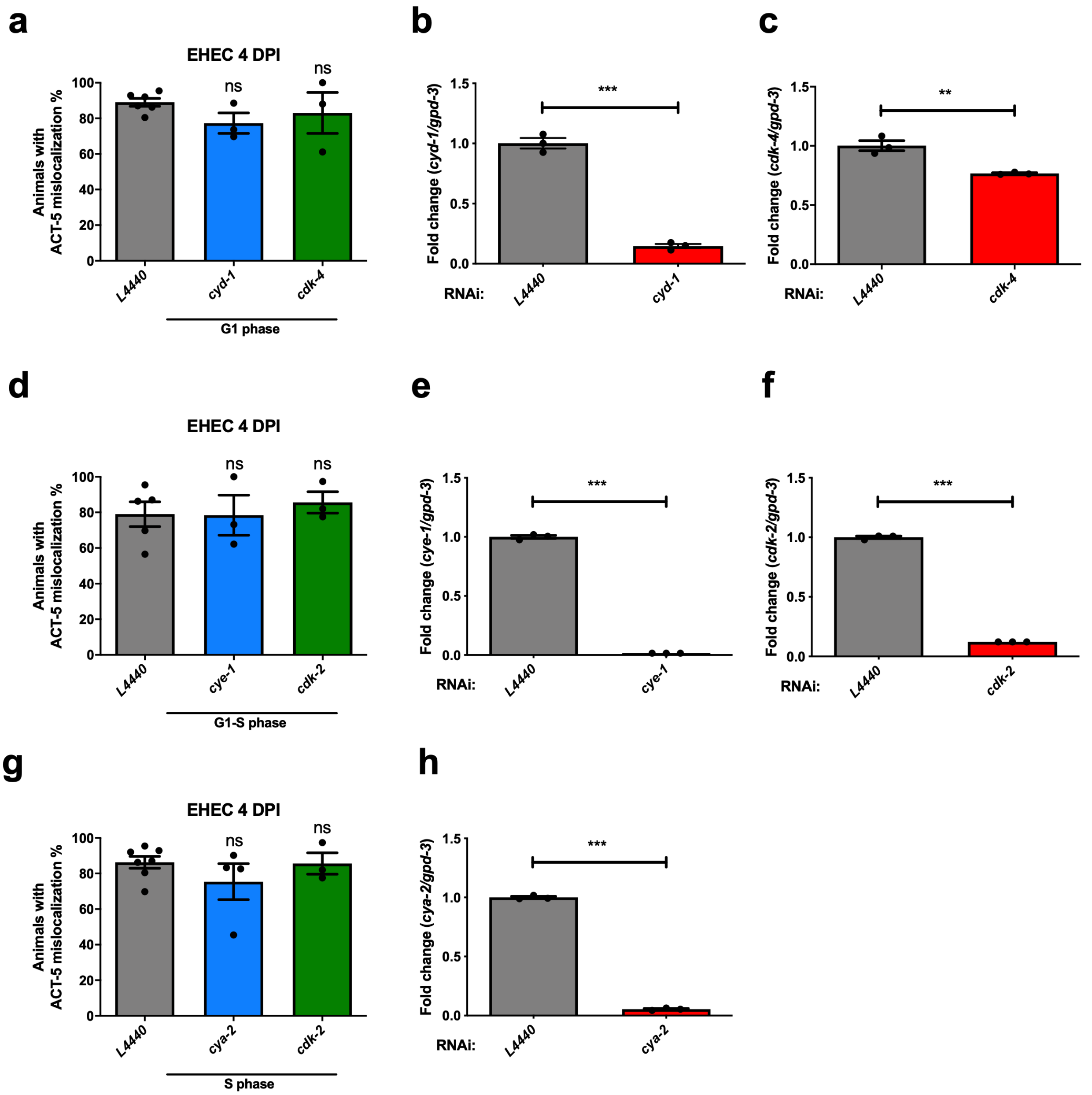

Fig. S2. Knockdown of the cell cycle G1, G1/S and S phase related genes by RNAi did not affect the EHEC-induced ACT-5 mislocalization in *C. elegans*. The percentage of ACT-5 mislocalization of *mCherry::ACT-5* animals treated RNAi of **(a)** the G1 phase: *cyd-1,* n=106, N=3; and *cdk-4,* n=83, N=3, **(d)** G1/S phase: *cye-1,* n=115, N=3; and *cdk-2,* n=106, N=3, and **(g)** S phase: *cya-2,* n=127, N=4; and *cdk-2,* n=106, N=3 related genes are similar to control L4440 group upon EHEC infection. ns indicates no significant difference by *t*-test. The q-RT-PCR analysis of mRNAs in the **(b)** *cyd-1* RNAi (N=3, P<0.0001) (**c**) *cdk-4* RNAi (N=3, P =0.0056) (**e**) *cye-1* RNAi (N=3, P<0.0001) (**f**) *cdk-2* RNAi (N=3, P<0.0001) (**h**) *cya-2* RNAi (N=3, P <0.0001) animals compared to L4440 RNAi control by *t*-test. All quantitative data are presented as mean±SEM, and each dot represented an independent result in the bar chart. All data statistics based on: * P<0.05, ** P<0.01, and *** P<0.001 by unpaired *t*-test (two-tailed).

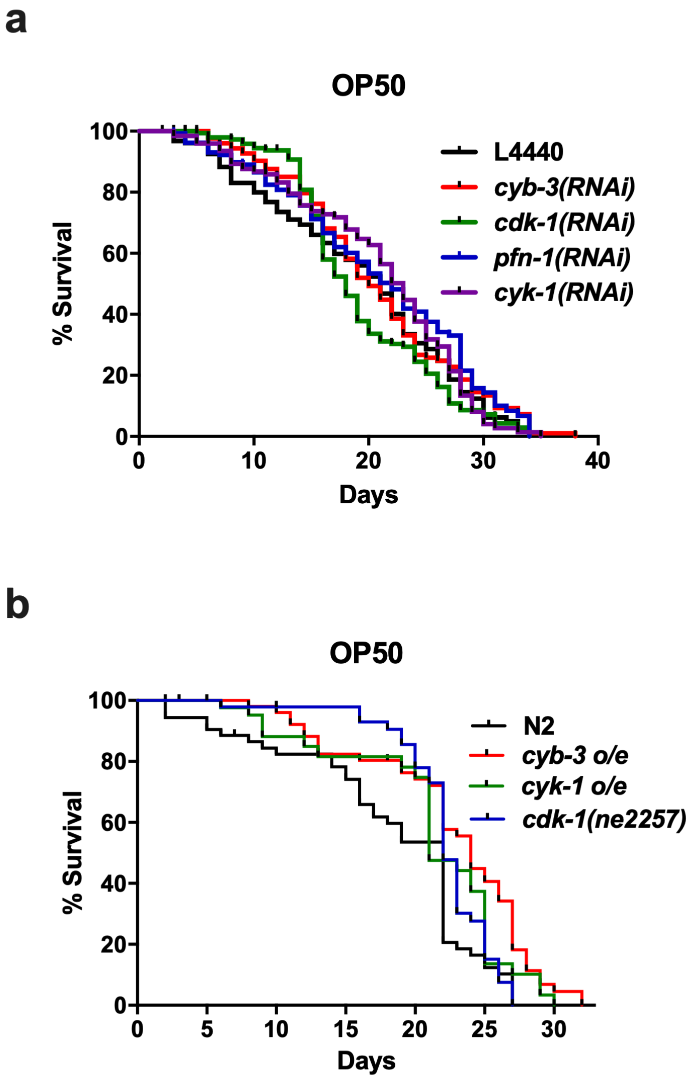

Fig. S3. The lifespan analysis of animals fed with OP50. (a) The survival curves of N2 animals fed with *cyb-3* RNAi (n=149, N=3, median survival days=20, and p=0.2436), *cdk-1* RNAi (n=162, N=3, median survival days=18, and *P*=0.4901), *pfn-1* RNAi (n=136, N=3, median survival days=22, and *P*=0.0911), *cyk-1* RNAi (n=127, N=3, median survival days=23, and *P*=0.3619) did not show decreased lifespan compared to L4440 RNAi control (n=156, N=3, median survival days=21). *P* values represent the results by the log-rank test. **(b)** The survival curves of *cyb-3 o/e* (n=153, N=3, median survival days=24, and *P*=0.0002) and *cyk-1 o/e* (n=142, N=3, median survival days=21, and *P*=0.0889) animals did not show decreased lifespan for OP50 treatment and the median survival day of *cdk-1(ne2257)* (n=152, N=3, median survival days=22, and *P*=0.0701) was similar compared to that of wild-type N2 (n=153, N=3, median survival days=22). *P* values represent the results by the log-rank test.

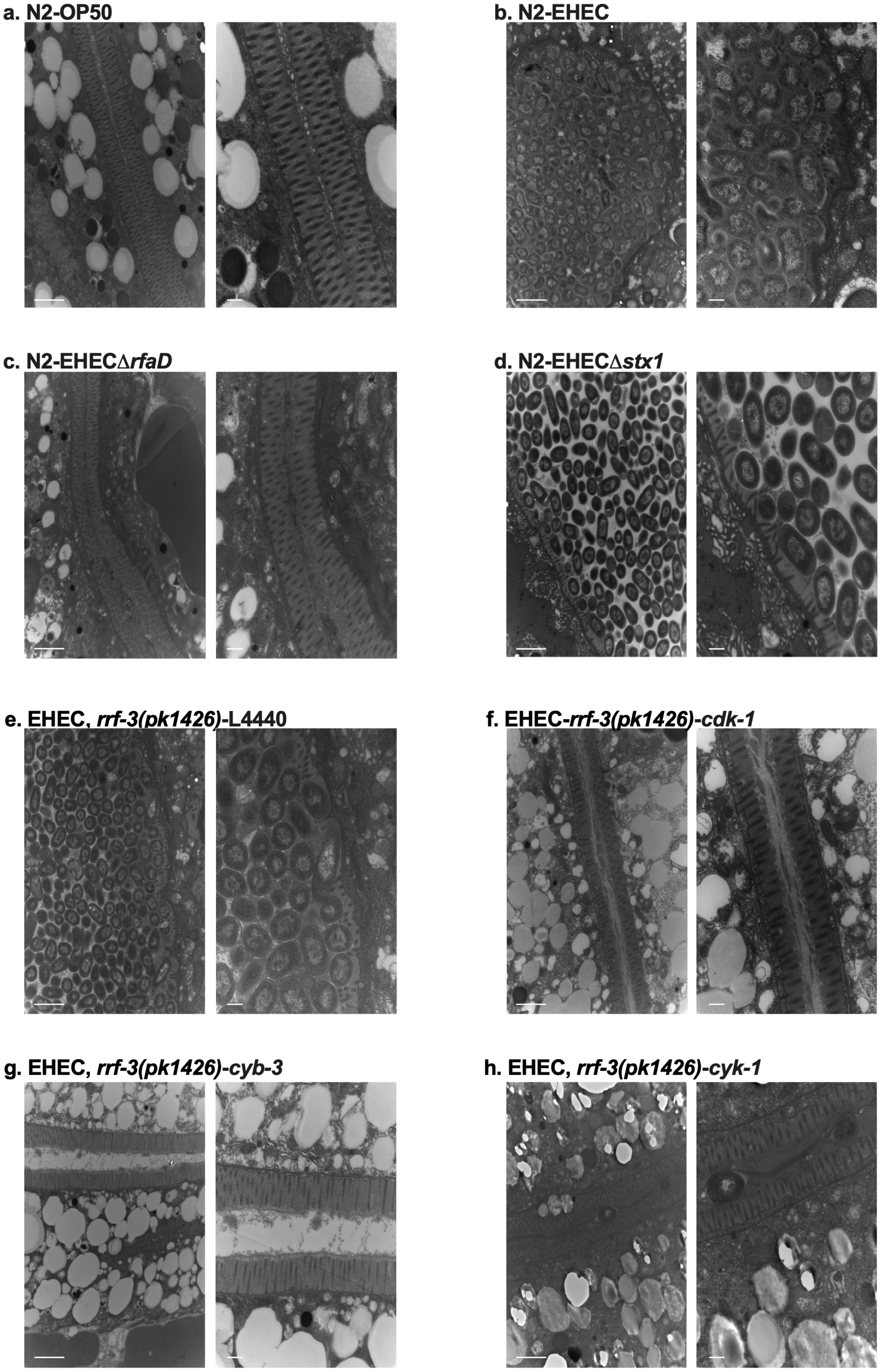

Fig. S4. TEM analysis of animals fed with OP50 or EHEC. T The represent TEM images of N2 animals fed with **(a)** OP50 (from ROI=5), **(b)** EHEC (from ROI=5 of each 228 μm^2 region), **(c)** EHEC *ΔrfaD* (from ROI=5 of each 228 μm^2 region), and **(d)** EHEC *Δstx1* (from ROI=5) for 4 days are shown. Scale bars in the left panels represent 2 μm, and in the right panels represent 500 nm. Moreover, the represent TEM images of the RNAi-sensitive *rrf-3(pk2426)* animals fed with EHEC and **(e)** the L4440 RNAi control (from ROI=5 of each 228 μm^2 region), **(f)** *cdk-1* RNAi (from ROI=5 of each 228 μm^2 region), **(g)** *cyb-3-1* RNAi ((from ROI=5 of each 228 μm^2 region), and **(h)** *cyk-1* RNAi (from ROI=5 of each 228 μm^2 region) for 4 days are shown. Scale bars in the left panels represent 2 μm, and in the right panels represent 500 nm. All TEM analysis were performed independently three times.

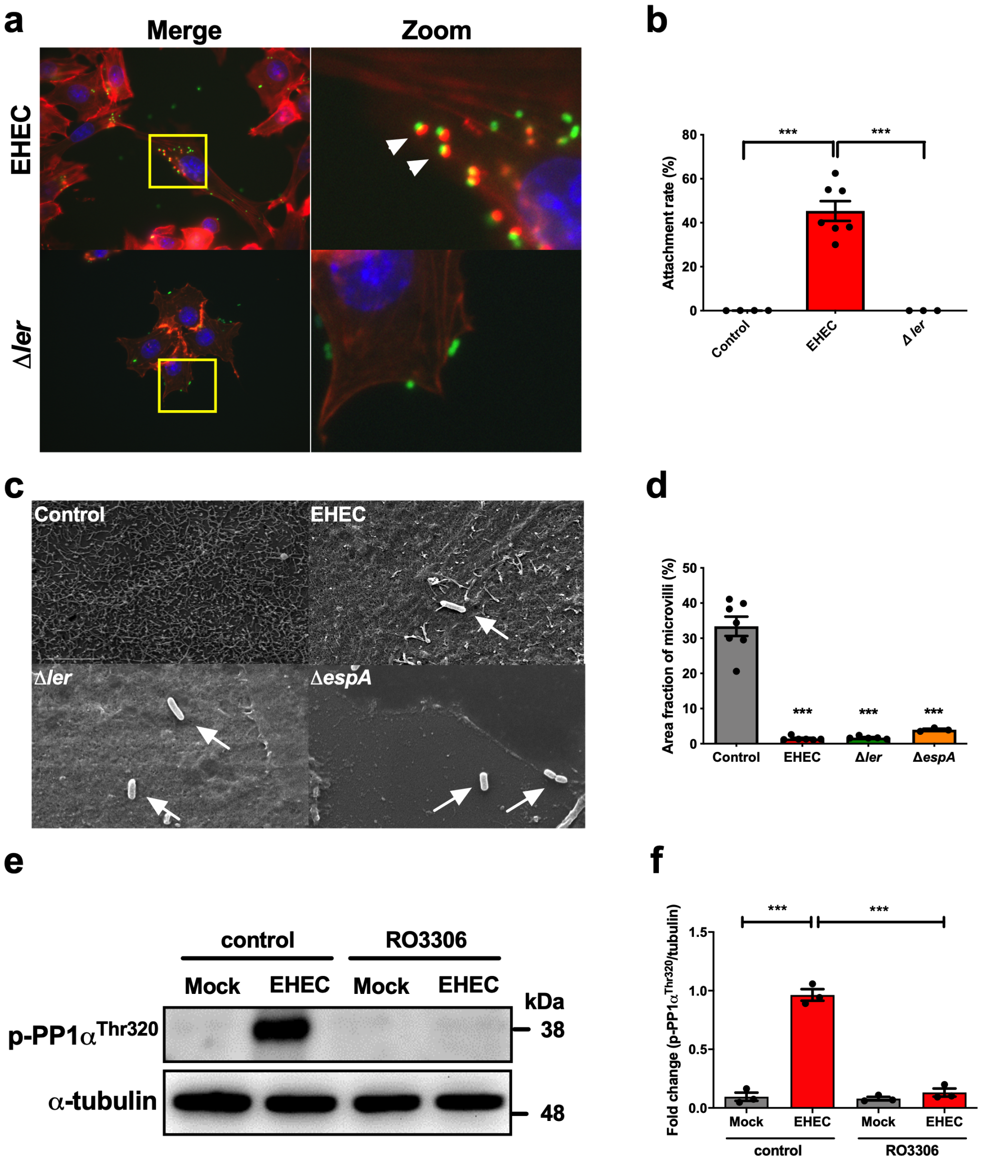

Fig. S5. T3SS does not involve in EHEC-induced microvillar effacement. (a) HeLa cells were infected by EHEC with the expression of GFP. Monolayers were examined microscopically after staining with BODIPY 558/568 phalloidin to detect F-actin and the nuclei were visualized by staining with DAPI. The pedestal formation and EHEC tightly attached to the HeLa cell, which EHEC (Green) and actin (Red) had colocalization, was observed in EHEC infected group but not in the *Δler* mutant infected group. **(b)** Quantitative analysis of the bacterial attachment rate observed by immunofluorescence microscopy. The attachment rate was significantly decreased in the *Δler* mutant compared to EHEC WT strain. *** represents *P*<0.001. **(c)** The SEM results showed that EHEC-induced microvillar effacement was not abolished in the EHEC *Δler* or *ΔespA* mutants. **(d)** The quantitative results reconfirmed that the area of microvilli was not increased post the infection by the *Δler* and *ΔespA* mutants compared to EHEC WT strain. *** represents *P*<0.001 compared to the control. Moreover, the efficacy of RO3306 treatment in Caco-2 cells was examined. **(e)** The represented western blot of p-PP1α^{Thr320} and α-tubulin (loading control) was shown. **(f)** The quantitative results of the western blot analysis in **S5e** was shown. Each dot in the bar charts represent the result of one independent experiment. N=3 for all groups. *** indicates statistically significant difference compared to the mock control by *t*-test. All quantitative data are presented as mean±SEM, and each dot represented an independent result in the bar chart. All data statistics based on: * *P*<0.05, ** *P*<0.01, and *** *P*<0.001 by unpaired *t*-test (two-tailed)

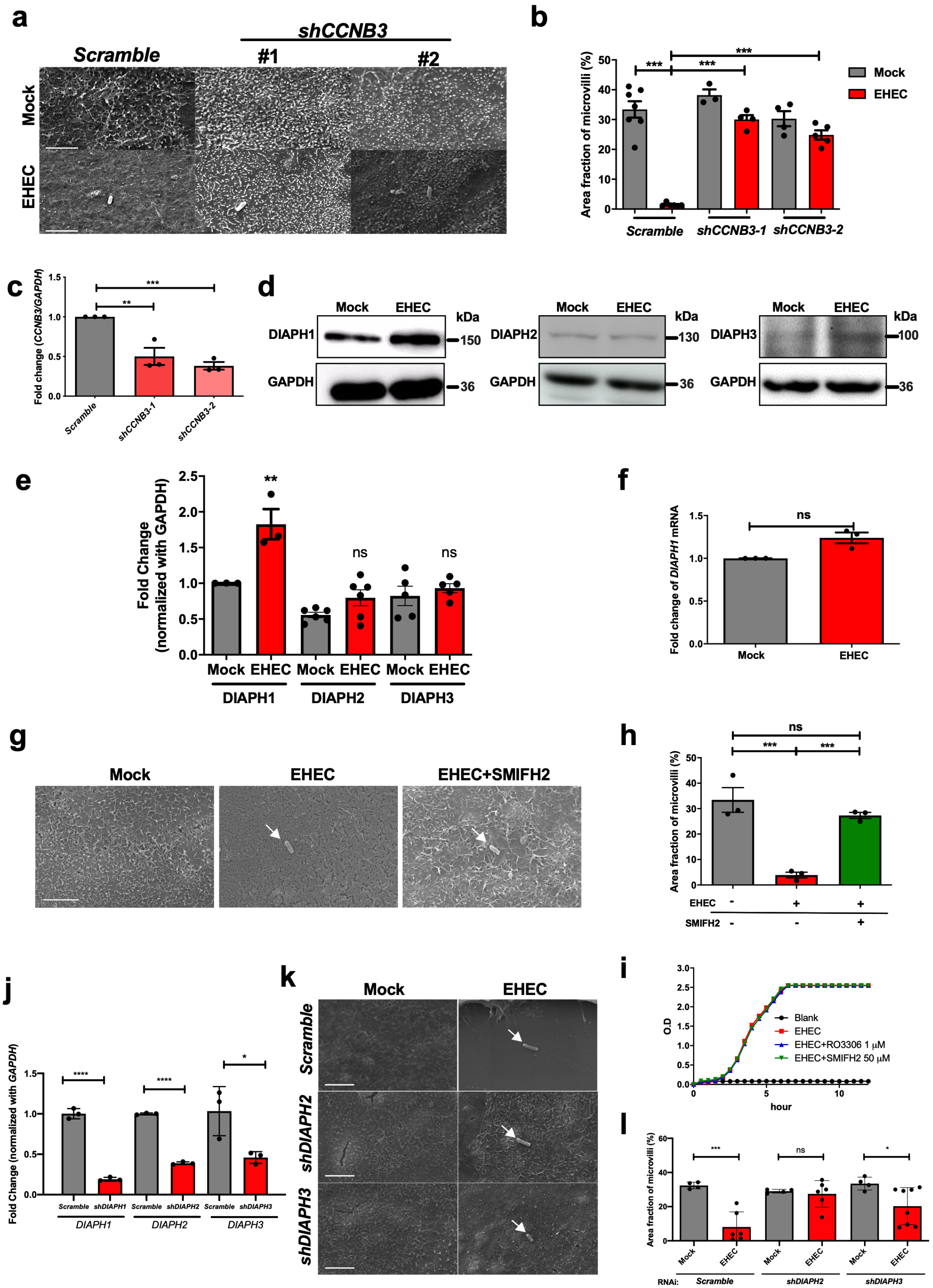
a
shCCNB3
Scramble
#1
#2
Mock
EHEC
b
Area fraction of microvilli (%)
Mock
EHEC
Scramble
shCCNB3-1
shCCNB3-2
c
Fold change (CCNB3/GAPDH)
Scramble
shCCNB3-1
shCCNB3-2
d
Mock
EHEC
kDa
DIAPH1
150
GAPDH
36
DIAPH2
130
DIAPH3
100
e
Fold Change (normalized with GAPDH)
DIAPH1
DIAPH2
DIAPH3
f
Fold change of DIAPH1 mRNA
g
Mock
EHEC
EHEC+SMIFH2
h
SMIFH2
j
Fold Change (normalized with GAPDH)
shDIAPH1
shDIAPH2
shDIAPH3
k
i
O.D
hour
Blank
EHEC
EHEC+RO3306 1 μM
EHEC+SMIFH2 50 μM
l
RNAi:

Fig. S6. *CCNB3* and *DIAPH1* are involved in the EHEC-induced micovillar effacement in Caco-2 cells. ((a) The represented SEM images (ROI=3 for each images) of Caco-2 cells infected by EHEC. Microvillar effacement was observed after EHEC infection in the control RNAi-treated (scramble) Caco-2 cells (EHEC, lower panel) compared to non-infected cells (Mock, upper panel). However, the EHEC-induced microvillar effacement was abolished in the *CCNB3* knockdown cells (*shCCBN3 #1* and *#2*) compared to the control RNAi-treated cells. Scale bars represent 5 µm. **(b)** The quantification of microvillar area in the experiments of S6a was shown. *** represents $P<0.0001$ by unpaired t-test (two-tailed). All experiment with ROI=3 of each 425 μm^2 region. **(c)** The knockdown efficacy of *shCCNBs* in Caco-2 cells was examined. Each dot represented an independent result in the bar chart. ** and *** represent $P=0.0099$ and $P=0.0002$, respectively by unpaired t-test (two-tailed). **(d)** The DIAPH1, DIAPH2, and DIAPH3 protein levels of Caco-2 cell infected EHEC for 0.5 hour were examined by western blotting. **(e)** The quantitative results showed that only the DIAPH1 protein level was increased by EHEC infection. Each dot represented an independent result in the bar chart. ** represents $P=0.0088$ by unpaired t-test (two-tailed). **(f)** qRT-PCR analysis of the expressions of *DIAPH1* mRNA from Caco-2 cell infected with EHEC. Each dot represented an independent result in the bar chart. ns represents no significance compared to control by t-test. **(g)** The representative SEM images of intestinal Caco-2 cell treated with mock control (Mock, ROI=3 of each 425 μm^2 region), EHEC wild type (EHEC, ROI=3 of each 425 μm^2 region), and EHEC with 50 µM of the pan-formin inhibitor SMIFH2 (EHEC+SMIFH, ROI=3 of each 425 μm^2 region). **(h)** The quantification of microvillar area in **S6g**. SMIFH2 significantly abolished the microvillar effacement induced by EHEC. *** represent $P=0.0001$ by unpaired t-test (two-tailed). All experiment with ROI=3 of each 425 μm^2 region. **(i)** The growth curve analysis of the EHEC cells treated with RO3306 or SMIFH2. Our results showed that RO3306 (1 µM) or SMIFH2 (50 µM) did not inhibit the growth of EHEC cells. **(j)** The knockdown efficacy of *shDIAPH, shDIAPH2,* and *shDIAPH3* in Caco-2 cells was examined. Each dot represented an independent result in the bar chart. * and **** represent $P=0.0334$ and $P<0.0001$, respectively by unpaired t-test (two-tailed)t. **(k)** The represented SEM images (ROI=4 of each 425 μm^2 region) of Caco-2 cells infected by EHEC and RNAi of *DIAPH1, DIAPH2,* or *DIAPH3.* **(l)** The quantification of microvillar area in **S6k**. *shDIAPH2* can significantly abolish the microvillar effacement induced by EHEC. * and *** represent $P=0.0428$ and $P=0.0007$, respectively by unpaired t-test (two-tailed). Each dot in the bar charts represent the result of one independent experiment. All experiment with ROI=4 of each 425 μm^2 region. All quantitative data are presented as mean±SEM, and each dot represented an independent result in the bar chart. All data statistics based on: * $P<0.05$, ** $P<0.01$, and *** $P<0.001$ by unpaired t-test (two-tailed)

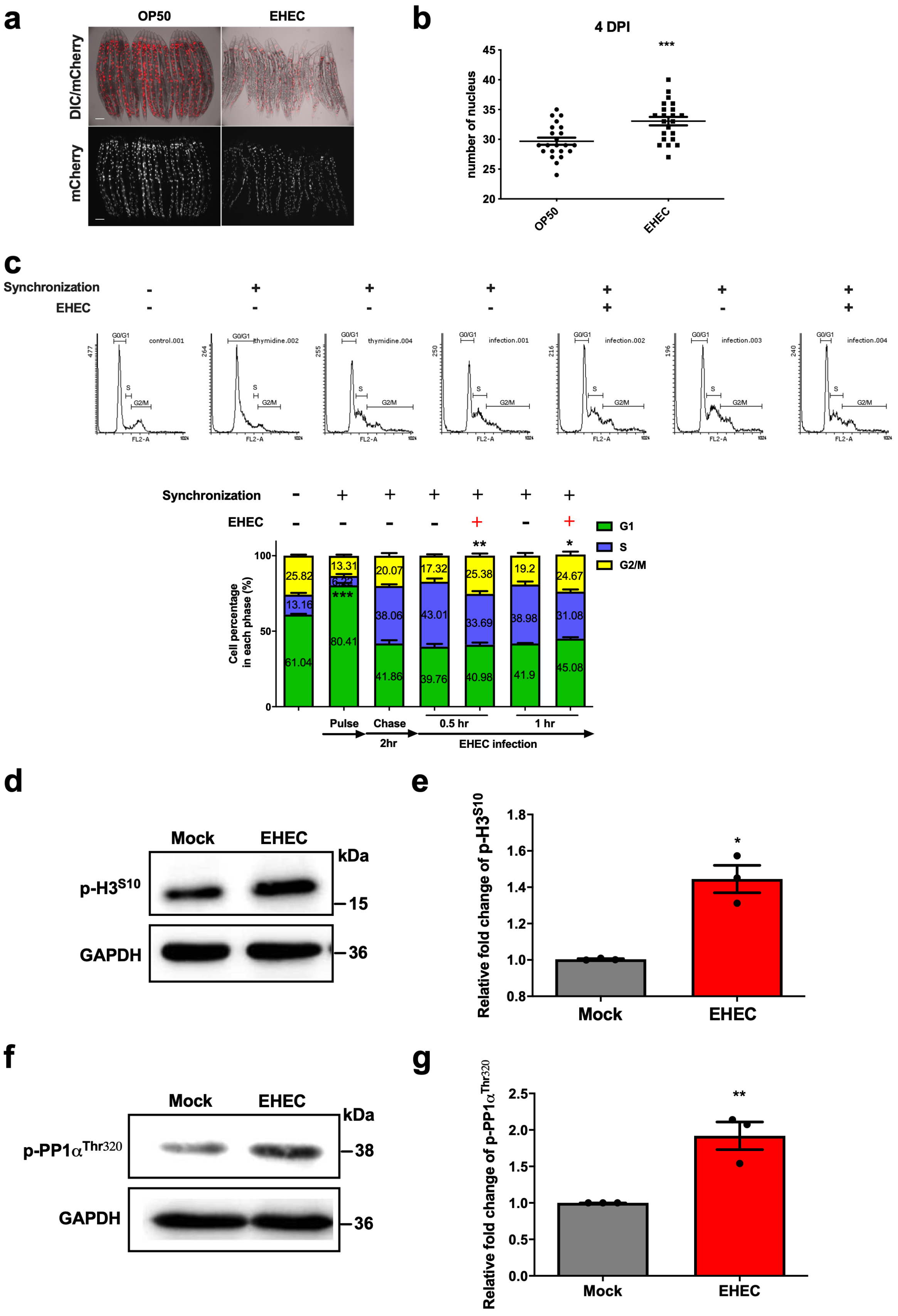
a
OP50
EHEC
DIC/mCherry
mCherry
b
4 DPI
***
number of nucleus
45
40
35
30
25
20
OP50
EHEC
c
Synchronization
EHEC
G0/G1
S
G2/M
FL2-A
control.001
thymidine.002
thymidine.004
infection.001
infection.002
infection.003
infection.004
G1
S
G2/M
Cell percentage in each phase (%)
100
50
0
25.82
13.16
61.04
13.31
6.22
80.41
***
20.07
38.06
41.86
17.32
43.01
39.76
**
25.38
33.69
40.98
19.2
38.98
41.9
*
24.67
31.08
45.08
Pulse
Chase
2hr
0.5 hr
1 hr
EHEC infection
d
Mock
EHEC
kDa
p-H3S10
GAPDH
15
36
e
Relative fold change of p-H3S10
1.8
1.6
1.4
1.2
1.0
0.8
*
Mock
EHEC
f
Mock
EHEC
kDa
p-PP1αThr320
GAPDH
38
36
g
Relative fold change of p-PP1αThr320
2.5
2.0
1.5
1.0
0.5
0.0
**
Mock
EHEC

Fig. S7. The G2/M phase signals were induced post EHEC infection. ((a) The *app-1p::mCherry-HistoneH2B* transgenic animals fed with OP50 or EHEC for four days. DIC/mCherry indicated the DIC images overlaid with the mCherry images. Each red dot indicated a nucleus in the intestinal cell. Scale bar represents 100 μm. **(b)** Quantification of the number of intestinal nuclei. After EHEC infection four days, the number of intestinal nuclei was significantly increased compared to OP50 group. OP50 group n=22 N=3, EHEC group n=22 N=3. Each dot represents the result of the number of intestinal nuclei of one worm. *** represents *P*=0.0008 by by unpaired *t*-test (two-tailed). **(c)** The cell cycle distribution was analyzed by the FlowJo software, which fits the best Gaussian distribution curve to each peak Go/G1, G2/M, and then calculated the S phase. The quantification of the results of flow cytometry analysis was also shown. After EHEC infection 0.5 to 1 hour, the population of G2/M phase were significantly increased compared to non-infection group. * and ** represent *P*=0.0388 and *P*=0.0029 by unpaired *t*-test (two-tailed). **(d)** Examination of the G2/M marker, phospho-$H3^{S10}$, in Caco-2 cell upon EHEC infection for 0.5 hour by western blotting. **(e)** Quantification of western blotting intensity showed that the expression of p-$H3^{S10}$ was significantly increased after EHEC infection. * represents P<0.05 by unpaired *t*-test (two-tailed). **(f)** Examination of phospho-PP1α protein level in Caco-2 cell upon EHEC infection for 0.5 hour by western blotting. **(g)** The quantification data of western blotting. The protein level of p-PP1α was increased during EHEC infection compared to non-infection group. ** represents *P*=0.0084 by unpaired *t*-test (two-tailed). Each dot in the bar charts represents the result of one independent experiment. All quantitative data are presented as mean±SEM, and each dot represented an independent result in the bar chart. All data statistics based on: * *P*<0.05, ** *P*<0.01, and *** *P*<0.001 by unpaired *t*-test (two-tailed).

References

1 Chou, T. C. *et al.* Enterohaemorrhagic Escherichia coli O157:H7 Shiga-like toxin 1 is required for full pathogenicity and activation of the p38 mitogen-activated protein kinase pathway in Caenorhabditis elegans. *Cell Microbiol* **15**, 82-97, doi:10.1111/cmi.12030 (2013).

2 Zarrilli, R. *et al.* Cell cycle block at G1-S or G2-M phase correlates with differentiation of Caco-2 cells: effect of constitutive insulin-like growth factor II expression. *Gastroenterology* **116**, 1358-1366, doi:10.1016/s0016-5085(99)70500-7 (1999).

3 Brenner, S. The genetics of Caenorhabditis elegans. *Genetics* **77**, 71-94 (1974).

4 Sato, M. *et al.* Caenorhabditis elegans SNAP-29 is required for organellar integrity of the endomembrane system and general exocytosis in intestinal epithelial cells. *Mol Biol Cell* **22**, 2579-2587, doi:10.1091/mbc.E11-04-0279 (2011).

5 Mi-Mi, L., Votra, S., Kemphues, K., Bretscher, A. & Pruyne, D. Z-line formins promote contractile lattice growth and maintenance in striated muscles of C. elegans. *J Cell Biol* **198**, 87-102, doi:10.1083/jcb.201202053 (2012).

6 Tarailo-Graovac, M. & Chen, N. Proper cyclin B3 dosage is important for precision of metaphase-to-anaphase onset timing in Caenorhabditis elegans. *G3 (Bethesda)* **2**, 865-871, doi:10.1534/g3.112.002782 (2012).

7 Los, F. C. *et al.* RAB-5- and RAB-11-dependent vesicle-trafficking pathways are required for plasma membrane repair after attack by bacterial pore-forming toxin. *Cell Host Microbe* **9**, 147-157, doi:10.1016/j.chom.2011.01.005 (2011).

8 Estes, K. A., Szumowski, S. C. & Troemel, E. R. Non-lytic, actin-based exit of intracellular parasites from C. elegans intestinal cells. *PLoS pathogens* **7**, e1002227-e1002227, doi:10.1371/journal.ppat.1002227 (2011).

9 Kang, J., Shin, D., Yu, J. R. & Lee, J. Lats kinase is involved in the intestinal apical membrane integrity in the nematode Caenorhabditis elegans. *Development* **136**, 2705-2715, doi:10.1242/dev.035485 (2009).

10 Ou, C.-Y. *et al.* Two cyclin-dependent kinase pathways are essential for polarized trafficking of presynaptic components. *Cell* **141**, 846-858, doi:10.1016/j.cell.2010.04.011 (2010).

11 Chen, H. D. *et al.* HLH-30/TFEB-mediated autophagy functions in a cell-autonomous manner for epithelium intrinsic cellular defense against bacterial pore-forming toxin in C. elegans. *Autophagy* **13**, 371-385, doi:10.1080/15548627.2016.1256933 (2017).

12 Strockbine, N. A. *et al.* Two toxin-converting phages from Escherichia coli O157:H7 strain 933 encode antigenically distinct toxins with similar biologic activities. *Infect Immun* **53**, 135-140 (1986).

13 Yu, S. L., Ko, K. L., Chen, C. S., Chang, Y. C. & Syu, W. J. Characterization of the distal tail fiber locus and determination of the receptor for phage AR1, which specifically infects Escherichia coli O157:H7. *J Bacteriol* **182**, 5962-5968, doi:10.1128/jb.182.21.5962-5968.2000 (2000).

14 Kudva, I. T., Hatfield, P. G. & Hovde, C. J. Characterization of Escherichia coli O157:H7 and other Shiga toxin-producing E. coli serotypes isolated from sheep. *J Clin Microbiol* **35**, 892-899 (1997).

15 Timmons, L. & Fire, A. Specific interference by ingested dsRNA. *Nature* **395**, 854, doi:10.1038/27579 (1998).

16 Kuo, C. J. *et al.* Mutation of the Enterohemorrhagic Escherichia coli Core LPS Biosynthesis Enzyme RfaD Confers Hypersusceptibility to Host Intestinal Innate Immunity In vivo. *Front Cell Infect Microbiol* **6**, 82, doi:10.3389/fcimb.2016.00082 (2016).
