# Supplementary material for: Host CDK-1 and formin mediate microvillar effacement induced by enterohemorrhagic Escherichia coli
Source: Nat Commun. 2021 Jan 4;12:90. doi: 10.1038/s41467-020-20355-1 (PMC7782584; doi:10.1038/s41467-020-20355-1)
Supplement: Supplementary file 3 — Description of Additional Supplementary Files [file 41467_2020_20355_MOESM3_ESM.pdf]

Description of Additional Supplementary Files

File Name: Supplementary Data 1
Description: The EHEC-response genes in C. elegans

File Name: Supplementary Data 2
Description: RNAi screen for the EHE-induced ACT-5 mislocalization phenotype
