# Supplementary material for: Host CDK-1 and formin mediate microvillar effacement induced by enterohemorrhagic Escherichia coli
Source: Nat Commun. 2021 Jan 4;12:90. doi: 10.1038/s41467-020-20355-1 (PMC7782584; doi:10.1038/s41467-020-20355-1)
Supplement: Supplementary file 6 — Reporting Summary [file 41467_2020_20355_MOESM6_ESM.pdf]

nature research

Corresponding author(s): Chang-Shi Chen

Last updated by author(s): Nov 16, 2020

Reporting Summary

Nature Research wishes to improve the reproducibility of the work that we publish. This form provides structure for consistency and transparency in reporting. For further information on Nature Research policies, see our Editorial Policies and the Editorial Policy Checklist.

Statistics

For all statistical analyses, confirm that the following items are present in the figure legend, table legend, main text, or Methods section.

n/a	Confirmed	
☐	☒	The exact sample size (*n*) for each experimental group/condition, given as a discrete number and unit of measurement
☐	☒	A statement on whether measurements were taken from distinct samples or whether the same sample was measured repeatedly
☐	☒	The statistical test(s) used AND whether they are one- or two-sided<br>*Only common tests should be described solely by name; describe more complex techniques in the Methods section.*
☒	☐	A description of all covariates tested
☐	☒	A description of any assumptions or corrections, such as tests of normality and adjustment for multiple comparisons
☐	☒	A full description of the statistical parameters including central tendency (e.g. means) or other basic estimates (e.g. regression coefficient) AND variation (e.g. standard deviation) or associated estimates of uncertainty (e.g. confidence intervals)
☐	☒	For null hypothesis testing, the test statistic (e.g. *F*, *t*, *r*) with confidence intervals, effect sizes, degrees of freedom and *P* value noted<br>*Give P values as exact values whenever suitable.*
☒	☐	For Bayesian analysis, information on the choice of priors and Markov chain Monte Carlo settings
☒	☐	For hierarchical and complex designs, identification of the appropriate level for tests and full reporting of outcomes
☒	☐	Estimates of effect sizes (e.g. Cohen's *d*, Pearson's *r*), indicating how they were calculated

Our web collection on statistics for biologists contains articles on many of the points above.

Software and code

Policy information about availability of computer code

Data collection	All confocal images were taken by the FV1000 Confocal Imaging System (Olympus)
Data analysis	Prism (version 6.0) was used for statistical analysis and generating graphys.<br>image J (version 1.8.0) was used for image quantitation.

For manuscripts utilizing custom algorithms or software that are central to the research but not yet described in published literature, software must be made available to editors and reviewers. We strongly encourage code deposition in a community repository (e.g. GitHub). See the Nature Research guidelines for submitting code & software for further information.

Data

Policy information about availability of data

All manuscripts must include a data availability statement. This statement should provide the following information, where applicable:
- Accession codes, unique identifiers, or web links for publicly available datasets
- A list of figures that have associated raw data
- A description of any restrictions on data availability

All datas supporting the results of this study are available within the paper and its supplemental Information.

Field-specific reporting

Life sciences study design

All studies must disclose on these points even when the disclosure is negative.

Sample size	No statistical methods were used to predetermine sample sizes for experiments. In our experiments, sample sizes were estimated based on previous literatures and were chosen in order to be able to perfrom statistical analyses, as is standard in the field.
Data exclusions	In the lifespan and ACT-5 mislocalization assay, worms that crawled off the plate were not includeed in the data.
Replication	All attempts at replication were successful.
Randomization	Worms and cells were chosen unbiasedly for experiments.
Blinding	The investigators were blinded to the genotype or RNAi treatments during the lifespan and ACT-5 mislocaliztion assay.

Reporting for specific materials, systems and methods

We require information from authors about some types of materials, experimental systems and methods used in many studies. Here, indicate whether each material, system or method listed is relevant to your study. If you are not sure if a list item applies to your research, read the appropriate section before selecting a response.

Materials & experimental systems

n/a	Involved in the study	
☐	☒	Antibodies
☐	☒	Eukaryotic cell lines
☒	☐	Palaeontology and archaeology
☐	☒	Animals and other organisms
☒	☐	Human research participants
☒	☐	Clinical data
☒	☐	Dual use research of concern

Methods

n/a	Involved in the study	
☒	☐	ChIP-seq
☐	☒	Flow cytometry
☒	☐	MRI-based neuroimaging

Antibodies

Antibodies used

1. Rabbit polyclonal anti-GFP antibody: Supplier/Abcam; Cat.No./ab6556; Lot.No./GR3271077-1; Dilution: 1/2500 for western blot.
2. Mouse monoclonal anti-α-Tubulin antibody: Supplier/Sigma Aldrich; Cat.No./T6199; Lot.No./029M4842V; Dilution: 1/2500 for western blot.
3. Rat monoclonal anti-SUN-1 (phospho-S43) antibody: Supplier/University of Vienna, Austria; Dilution: 1/1000 for western blot.
4. Rabbit monoclonal anti-GAPDH antibody: Supplier/Abcam; Cat.No./ab181602; Lot.No./GR217575-37; Dilution: 1/2500 for western blot.
5. Rabbit polyclonal anti-Histone H3 (phospho S10) antibody: Supplier/Abcam; Cat.No./ab47297; Lot.No./GR45154-14; Dilution: 1/1000 for western blot.
6. Rabbit monoclonal anti-DIAPH1 antibody: Supplier/Cell Signaling; Cat.No./14634; Lot.No./1; Dilution: 1/1000 for western blot.
7. Rabbit polyclonal anti-DIAPH2 antibody: Supplier/Cell Signaling; Cat.No./5474; Lot.No./1; Dilution: 1/1000 for western blot.
8. Mouse polyclonal anti-DIAPH3 antibody: Supplier/Sigma Aldrich; Cat.No./SAB1409850; Lot.No./K6041; Dilution: 1/1000 for western blot.

Validation

All antibodies used in this study were validated by the supplier, the information was available on their websites.

Eukaryotic cell lines

Policy information about cell lines

Cell line source(s)	Hela cells (ATCC, CCL-2) and Caco-2 cells (ATCC, CRL-2102) cell lines were ordered from the cell bank of American Type Culture Collection
Authentication	The cell line has been validated using the short tandem repeat (STR) profiling method by the cell bank
Mycoplasma contamination	Not tested
Commonly misidentified lines (See ICLAC register)	No commonly misidentified lines were used

Animals and other organisms

Policy information about studies involving animals; ARRIVE guidelines recommended for reporting animal research

Laboratory animals	C. elegans strains: The wild-type Bristol N2, GK454 (unc-119(ed3);dkIs247), DWP13 (unc-119(ed4);upsIs3), JNC100 (unc-119 (ed3);dotSi100), GK288 (unc-119(ed3);dkI5166), ERT38 (kcIs6;caIs38), WM99 (cdk-1(ne2257)), YQ203 (unc-119(ed3);wfIs52), YQ387 (unc-119(ed3);dotSi100;dkIs247), YQ388 (unc-119(ed3);wfEx338;dkIs247), YQ389 (pfn-1(ok808); unc-119(ed3);dkIs247), YQ420 (unc-119(ed4);upsIs3;dkIs247), YQ422 (cdk-1(ne2257);unc-119(ed3);dkIs247), YQ486 (unc-119(ed3);dkIs247;wfEx388), YQ487 (unc-119(ed3);dkIs247;wfEx392), YQ488 (unc-119(ed3);dkIs247;wfEx391), YQ489 (unc-119(ed3);dkIs247;wfEx390), YQ490 (unc-119 (ed3);dkIs247;wfEx396), YQ494 (unc-119(ed4);upsIs3;dkIs247;wfEx404), YQ525 (unc-119(ed3);dkIs247;wfEx388), YQ526 (unc-119 (ed3);dkIs247;wfEx390), and YQ527 (unc-119(ed3);dkIs247;wfEx396)
Wild animals	This study did not invovled wild animals
Field-collected samples	Not invovle
Ethics oversight	In the C. elegans experiments, ethics oversight is not required.

Note that full information on the approval of the study protocol must also be provided in the manuscript.

Flow Cytometry

Plots

Confirm that:

- [x] The axis labels state the marker and fluorochrome used (e.g. CD4-FITC).
- [x] The axis scales are clearly visible. Include numbers along axes only for bottom left plot of group (a 'group' is an analysis of identical markers).
- [x] All plots are contour plots with outliers or pseudocolor plots.
- [x] A numerical value for number of cells or percentage (with statistics) is provided.

Methodology

Sample preparation	Caco-2 cells were seeded at a density of 30,000 cells/6-well plate in Dulbecco's modified Eagle medium with 10% fetal bovine serum. On the 3rd day of culture, cells were synchronized by double-thymidine block as described previously2. Cells were grown in 2 mM thymidine for 18 hours in a cell incubator, and then released into fresh medium without thymidine (Sigma Aldrich, 50-89-5) for 9 hours. Next, cells were incubated in the presence of 2 mM thymidine for 12 hours. After discarding the thymidine-containing medium, cells were released into fresh DMEM medium and synchronized at the G1 phase. Before infection, cells were incubated at 37C for 2 hours to enrich the S phase population. When cells were synchronized at S phase, cells were infected with EHEC EDL933 immediately with the MOI: EHEC and Caco-2 cells at 50:1 for 0.5 to 1 hour. After infection, cells were washed twice by PBS and detached from plates by using a trypsin-EDTA solution. Cells lysate were moved to a 15 mL tube and centrifuged at 2,000 × g for 3 minutes. The supernatant was then removed and 3 mL 70% ethanol was added to the tube. The samples were stored at -20C. On the day of flow cytometry analysis, the sample was centrifuged at 2,000 × g for 3 minutes and supernatant was then removed. Mixed reagent (1 mL; 20 µL Propidium iodide + 20 µL 5% Triton X-100 + 20 µL 1 mg/mL RNase A +940 µL PBS) was added to the tube and the sample was passed through a cell filter before analysis. Cell cycle phases analysis was conducted.
Instrument	FACSCalibur flow cytometer (BD Biosciences)
Software	Cy esprit flowway 2.3
Cell population abundance	When cells were sorted or enriched, the purity was confirmed by flow cytometry and in each case was above 90% purity.
Gating strategy	Gate on the single cell population using pulse width vs. pulse area. Then apply this gate to the scatter plot and gate out obvious debris. Combine the gates and apply to the PI histogram plot.

- [x] Tick this box to confirm that a figure exemplifying the gating strategy is provided in the Supplementary Information.
